# The association between iris color and refractive errors in children

**DOI:** 10.1038/s41598-024-52807-9

**Published:** 2024-01-25

**Authors:** Mehrdad Ebrahimi, Anneh Mohammad Gharravi, Roqayeh Aliyari, Mohammad Hassan Emamian, Mehdi Khabazkhoob, Hassan Hashemi, Akbar Fotouhi

**Affiliations:** 1 Student Research Committee, School of Medicine, Shahroud University of Medical Sciences, Shahroud, Iran; 2 School of Medicine, Shahroud University of Medical Sciences, Shahroud, Iran; 3 Ophthalmic Epidemiology Research Center, Shahroud University of Medical Sciences, Shahroud, Iran; 4 Department of Medical Surgical Nursing, School of Nursing and Midwifery, Shahid Beheshti University of Medical Sciences, Tehran, Iran; 5 Noor Research Center for Ophthalmic Epidemiology, Noor Eye Hospital, Tehran, Iran; 6 Department of Epidemiology and Biostatistics, School of Public Health, Tehran University of Medical Sciences, Tehran, Iran

**Keywords:** Epidemiology, Eye diseases

## Abstract

This study aimed to evaluate the association between iris color and refractive errors in children aged 6–12 years. This cross-sectional study was based on data obtained from the first phase of the Shahroud Schoolchildren Eye Cohort Study. The target population was 6 to12 year-old students living in urban and rural areas. Iris colors were classified by comparing eye colors with close-up images of iris colors. Myopia was defined as a spherical equivalent (SE) ≤ − 0.5 diopter and hyperopia was defined as SE ≥ 2 diopter in cycloplegic refraction. The association of iris color with hyperopia and myopia was investigated by fitting two separate multiple logistic regression models adjusted for place of residence, age, sex, and times for outdoor activity and near work. Among the 5394 participates with the mean age of 9.7 year, the prevalence of myopia and hyperopia was 4.8% and 4.7% respectively. The number and proportion (in parentheses) of amber, light blue, light brown, dark brown, gray, green and hazel iris colors were 19(0.4%), 26(0.5%), 645(12.0%), 4517(83.7%), 4(0.1%), 59(1.1%), and 124(2.3%) respectively. Compared to dark brown, the odds ratios and 95% confidence intervals (in parentheses) of myopia were 4.8(1.2–18.7), 0.8(0.1–5.8), 1.0(0.7–1.5), 0.4(0.1–2.7) and 0.6(0.2–1.8) for amber, light blue, light brown, green and hazel iris colors in multiple logistic regression model. No significant association was observed between iris colors and hyperopia. This study shows that amber iris is significantly associated with higher odds of myopia. These children should be further monitored and examined. More studies with higher sample size in all iris colors are recommended.

## Introduction

Iris color is a polymorphism and polygenic trait that varies according to race and ethnicity. The most common iris color in the world is brown, accounting for about 79%^1^. The association between iris color and eye diseases has been investigated in several studies^2–4^. Iris color is considered a protective factor for some diseases^4,5^. Besides, environmental factors are interactive with iris color and may affect eye growth and myopia^6^. Due to the association between iris color and some eye diseases, iris color can be used as a predictive factor in these diseases. For instance, ocular melanoma, a life-threatening disease, is more prevalent among people with blue and green eyes; therefore, being aware of this association can help in early diagnosis and better prognosis of this disease^7,8^.

Numerous studies have concluded that outdoor activities by exposing children to natural light, reduce the risk of myopia and play a protective role against myopia^9–11^. Also, myopia and increased axial length of the eye were more common in dark eyes than in light eyes in Chinese children^12^. In an Iranian study, iris color was not associated with myopia and the odds of hyperopia in dark and light brown were lower than green and yellow iris colors^13^.

Other eye diseases have also been linked to the iris color. For instance, some studies have suggested an association between Intraocular pressure (IOP) and iris color and reported a high prevalence of glaucoma in dark eyes^14,15^. However, in the Tehran Eye Study, the highest and lowest IOPs were observed in blue and green/yellow eyes, respectively^16^. In the Blue Mountains Eye Study, a significant association was observed between dark brown iris color and age-related cataract^17^. In several other studies, dark iris color has been reported as a risk factor for cataract^5,17–19^. On the other hand, the risk of age-related macular degeneration is more reported in people with light eye color^3,20^. In the Tehran Eye study, light brown iris color had the highest risk of age-related macular degeneration compared to other iris colors^21^.

Due to the high and increasing prevalence of refractive errors, especially in children, and contradictory findings regarding their association with iris colors, this study evaluated the association between iris colors and refractive errors in a sample of Iranian children aged 6 to12 years old.

## Methods

This cross-sectional study comes from the first phase of Shahroud Schoolchildren Eye Cohort Study^22^ which was designed to identify the causes of visual impairment and refractive errors in children. The first phase of this study was conducted in 2015. In this study, the target population was 6 to 12-year-old primary school children living in urban and rural areas in Shahroud located in the northeastern of Iran. Due to the small number of students living in rural areas, the total population of rural primary school students was selected, while in urban areas, students were selected using random cluster sampling. Classrooms were considered as clusters and 200 classrooms were randomly selected from 473 classrooms. In total 6624 students were invited and 5620 students (84.8%) participated in the study.

After explaining the study method, written informed consent was obtained from the students’ parents, and after interviewing them, eye examination and anthropometric measurements were performed for the participants. For all students, a complete optometric examination, iris color determination, and best-corrected and uncorrected visual acuity were measured, and glasses were prescribed if necessary. Eye biometry was performed with the Allegro BioGraph 900. Two trained optometrists performed examinations under standard conditions. To measure visual acuity, uncorrected visual acuity was first measured from a distance of 3 m using a Nidek CP-770 projector. Then, non-cycloplegic refraction was performed using Nidek ARK-510A autorefractokeratometer (Nidek Co. Ltd, Gammagori, Aichi, Japan). After refining the results by retinoscopy with Heine Beta 200 (HEINE Optotechnic, Hersching, Germany), best corrected visual acuity was measured and subjective refraction was performed. Finally, cycloplegic refraction and retinoscopy were performed at least 30 min after instilling 2 drops of 1% cyclopentolate with an interval of 5 min. Cycloplegia was investigated after 30 min by examining the pupil response to light and the extent of mydriasis, and if necessary, the third drop of cyclopentolate was used. Myopia was defined as a spherical equivalent on cycloplegic refraction equal to or lower than − 0.5 diopter. Hyperopia was defined as a spherical equivalent of + 2.0 diopter or higher.

**Figure 1:**
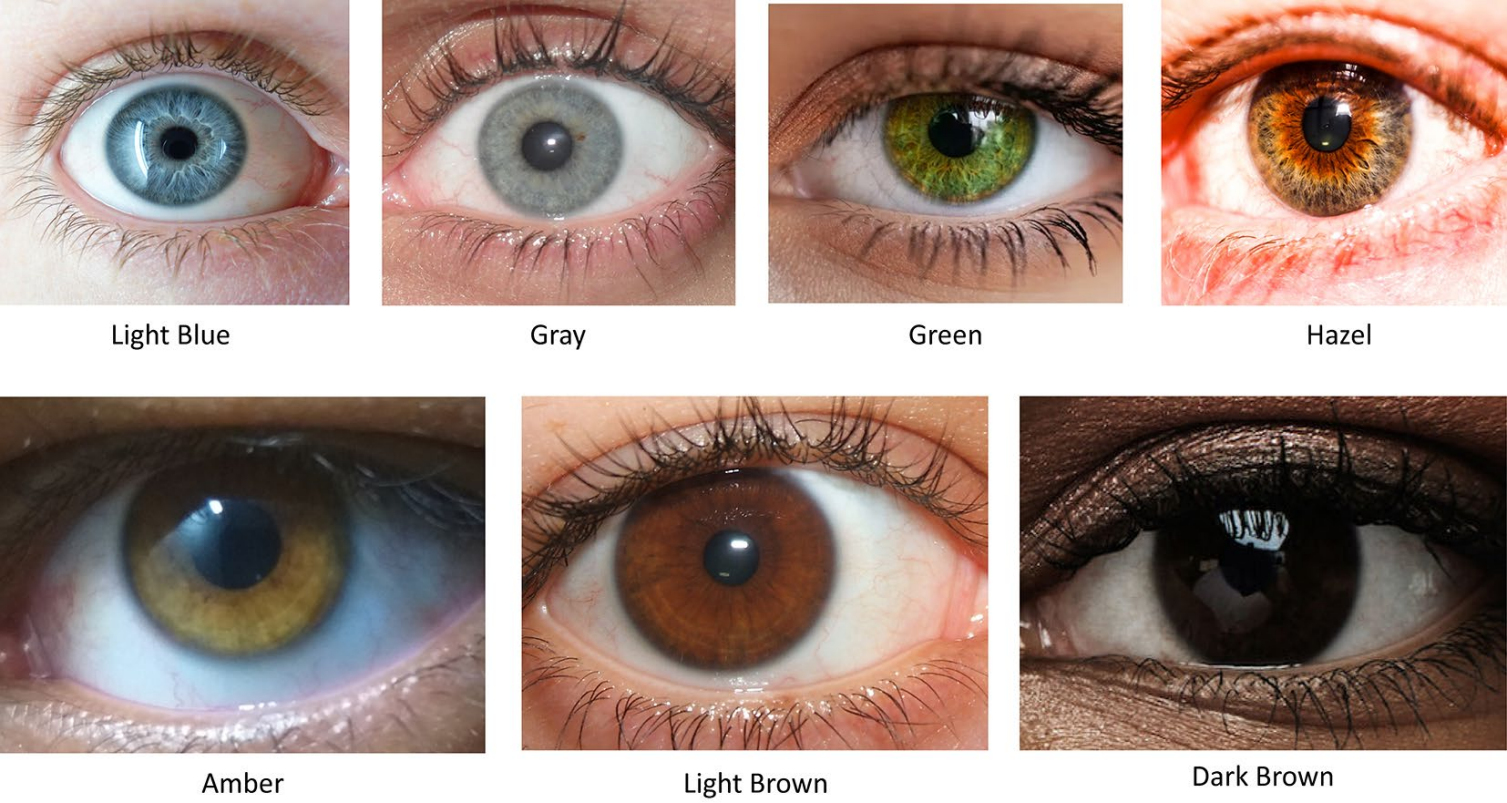
Examples of Iris colors, used for classification of participants. Pictures are taken from Wikipedia, the free encyclopedia (https://en.wikipedia.org/w/index.php?title=Eye_color&oldid=1187612730).

Although attempts have been made to introduce a standard method for iris color grading^23–25^, to the best of our knowledge, there has been no classification scale, approved by the scientific communities^26^. For this reason, we also prepared close-up images based on the colors of the iris that exist in Iranian people (Fig. 1). Different iris colors were described as follows: Amber: A golden or yellowish-brown color; Light Blue: A pale blue color; Light Brown: A warm, light brown color; Dark Brown: A rich, deep brown color; Gray: A cool, grayish color; Green: A greenish color; Hazel: A mixed color with hints of brown, green, and gold.

We assessed iris color on non-dilated eyes. Participants' iris color was subjectively divided into amber, blue, light brown, dark brown, gray, green, and hazel by two examiners and by comparing the eye colors with the standard color images. Considering that some studies recommended three groups for iris classification^23,25^ and to minimize any misclassification, we developed these new groups by combining amber, light brown and dark brown as Brown, green and hazel as Green, and blue and gray as Blue.

Due to the high correlation between the right and left eyes in terms of iris color, the participants' right eyes were analyzed. The relationship between the independent variables of eye color, residence place, age, and sex with hyperopia and myopia was investigated in two separate logistic regression models. Gray eye color has been disregarded in the logistic regression model due to sparse data. The mean and standard deviation of the axial length were compared in different iris color groups and statistically analyzed by analysis of variances (ANOVA) test. The effect of cluster sampling and sampling weight was considered in the calculation of standard error.

### Ethics approval statement

All methods of the study were carried out by relevant guidelines and regulations. The protocol of the study was approved by the Ethics Committee of Shahroud University of Medical Sciences (Ethics code: 100/108054). Students participate in the study willingly. Students’ parents signed written informed consent.

## Results

**Figure 2:**
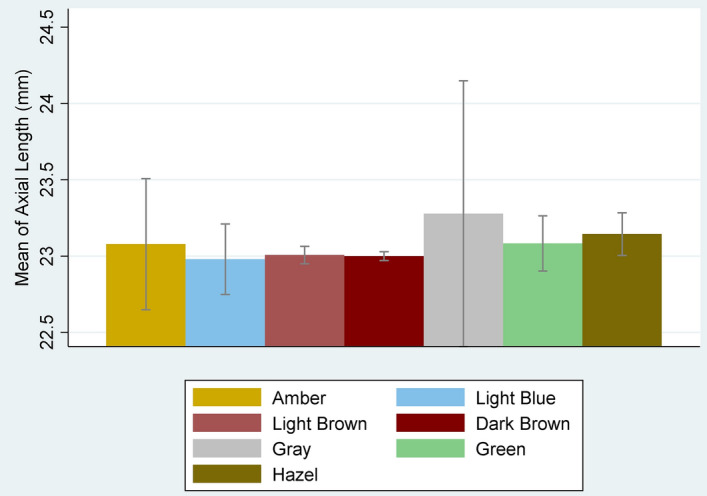
The mean and its 95% confidence intervals of axial length by iris colors.

Of the 5620 participants of cohort study, information on cycloplegic refraction and iris color of the right eyes was available for 5394 of them, so this study was performed on these individuals. In total, 53.0% of the participants were boys, and 19.5% of them lived in the rural area. The age of the individuals was between 6 and 12 years, with an average of 9.7 year. The standard deviation for mean age was 1.7 year. The description of refractive errors, axial length, spherical equivalent and iris colors by demographic variables is presented in Table 1. Overall, the prevalence of myopia (4.8%) and hyperopia (4.7%) was low. The mean axial length of the eye in all participants was 23.00 mm with a standard deviation of 0.95 mm. Dark brown had the highest frequency (83.7%) among the different iris colors. Gray eye color had the lowest frequency (4 people) and hyperopia or myopia was not observed in these students. Only 19 people had amber eye color (Table 2). Table 2 also shows the prevalence of myopia and hyperopia by different iris colors. Analysis of covariance showed that the mean spherical equivalent did not differ significantly between different iris colors after adjustment to age, sex, and place of residence (*P* = 0.267). The axial length was not significantly different in children with different iris colors (*P* = 0.720) (Fig. 2). The odds of myopia was 4.8 (95% CI 1.2–18.7) times higher in children with amber iris color compared to children with dark brown iris color after adjusting for sex, age, and residence place and times for outdoor activity and near work. Since high myopia is mainly related to genetic factors, in a sensitivity analysis, 9 cases of high myopia (cases with spherical equivalent ≤ -6) were excluded from the analysis, but the same results were found (Table 3).

**Table 1:** The description of refractive errors, axial length (AL), spherical equivalent (SE) and iris colors by demographic variables. Shahroud, Iran, 2015.

Demographic variables		N (%)	SE, Right eye (Mean ± SD)	AL, Right eye (Mean ± SD)	Myopia, n (%)*	High myopia, n	Hyperopia, n (%)*	Emmetropia, n (%)*	Iris colors (n (%)*)						
									Amber	Light Blue	Light Brown	Dark Brwon	Gray	Green	Hazel
Age groups (year)	6	218 (4.0)	1.0 ± 0.8	22.7 ± 0.8	5 (2.3)	0	16 (7.3)	197 (90.4)	1 (0.5)	0 (0)	18 (8.3)	196 (89.9)	0 (0)	2 (0.9)	1 (0.5)
	7	819 (15.2)	1.0 ± 0.7	22.7 ± 0.7	15 (1.8)	0	55 (6.7)	749 (91.5)	1 (0.1)	5 (0.6)	68 (8.3)	718 (87.7)	2 (0.2)	7 (0.9)	18 (2.2)
	8	974 (18.1)	0.9 ± 0.9	22.8 ± 1.2	37 (3.8)	1	48 (4.9)	889 (91.3)	4 (0.4)	3 (0.3)	111 (11.4)	829 (85.1)	1 (0.1)	12 (1.2)	14 (1.4)
	9	1024 (19.0)	0.9 ± 0.9	23.0 ± 0.8	49 (4.8)	1	61 (6.0)	914 (89.3)	5 (0.5)	3 (0.3)	128 (12.5)	859 (83.9)	0 (0)	7 (0.7)	22 (2.2)
	10	853 (15.8)	0.8 ± 0.8	23.1 ± 1.3	32 (3.8)	0	29 (3.4)	792 (92.9)	2 (0.2)	7 (0.8)	110 (12.9)	698 (81.8)	1 (0.1)	12 (1.4)	23 (2.7)
	11	894 (16.6)	0.7 ± 1.2	23.3 ± 0.8	61 (6.8)	5	35 (3.9)	798 (89.3)	3 (0.3)	5 (0.6)	127 (14.2)	721 (80.7)	0 (0)	9 (1.0)	29 (3.2)
	12	612 (11.4)	0.5 ± 1.0	23.4 ± 0.8	57 (9.3)	2	11 (1.8)	544 (88.9)	3 (0.5)	3 (0.5)	83 (13.6)	496 (81.1)	0 (0)	10 (1.6)	17 (2.8)
Sex	Male	2857 (53.0)	0.8 ± 0.9	23.2 ± 1.1	124 (4.3)	4	121 (4.2)	2612 (91.4)	10 (0.4)	16 (0.6)	356 (12.5)	2358 (82.5)	3 (0.1)	36 (1.3)	78 (2.7)
	Female	2537 (47.0)	0.8 ± 1.0	22.7 ± 0.7	132 (5.2)	5	134 (5.3)	2271 (89.5)	9 (0.4)	10 (0.4)	289 (11.4)	2159 (85.1)	1 (0)	23 (0.9)	46 (1.8)
Residence Place	Urban	4341 (80.5)	0.8 ± 0.9	23.0 ± 0.9	227 (5.2)	9	207 (4.8)	3907 (90.0)	11 (0.3)	25 (0.6)	489 (11.3)	3663 (84.4)	4 (0.1)	47 (1.1)	102 (2.4)
	Rural	1053 (19.5)	0.9 ± 1.0	22.9 ± 1.1	29 (2.8)	0	48 (4.6)	976 (92.7)	8 (0.8)	1 (0.1)	156 (14.8)	854 (81.1)	0 (0)	12 (1.1)	22 (2.1)
Total population		5394 (100)	0.8 ± 0.9	23.0 ± 1.0	256 (4.8)	9	255 (4.7)	4883 (90.5)	19 (0.4)	26 (0.5)	645 (12.0)	4517 (83.7)	4 (0.1)	59 (1.1)	124 (2.3)

*Percents calculated by rows. Myopia, hyperopia and high myopia were defined as SE ≤ − 0.5, SE ≥ 2.0, and SE ≤ − 0.6 diopter respectively.

**Table 2:** Iris color in right eyes by refractive errors in Iranian 6 to 12 years old children.

Iris color	N (%)	Spherical equivalent (Mean ± SD)	Myopia (%)	Hyperopia (%)
Amber	19 (0.4)	0.35 ± 1.60	3 (1.3)	1 (0.5)
Light Blue	26 (0.5)	1.00 ± 0.79	1 (0.4)	2 (1.0)
Light Brown	645 (12.0)	0.84 ± 0.98	29 (12.3)	28 (13.4)
Dark Brown	4517 (83.7)	0.79 ± 0.92	198 (83.9)	172 (82.3)
Gray	4 (0.1)	1.22 ± 0.23	0	0
Green	59 (1.1)	0.95 ± 0.78	1 (0.4)	3 (1.4)
Hazel	124 (2.3)	1.02 ± 1.06	4 (1.7)	3 (1.4)
Total	5394 (100)	0.80 ± 0.94	236 (100)	209 (100)

**Table 3:** The Association Between refractive errors and Iris Color in Multiple Logistic Regression Models.

Independent variables		Myopia		Hyperopia	
		(Odds ratio (95% CI))	*P* value	(Odds ratio (95% CI))	*P* value
Iris color	Dark Brown	Reference group	–	Reference group	–
	Light Brown	1.02 (0.69–1.51)	0.936	1.29 (0.85–1.96)	0.230
	Amber	4.79 (1.23–18.70)	0.024	0.87 (0.11–6.85)	0.898
	Light Blue	0.81 (0.11–5.76)	0.833	2.36 (0.54–10.30)	0.251
	Green	0.36 (0.05–2.73)	0.321	1.47 (0.41–5.29)	0.554
	Hazel	0.62 (0.21–1.82)	0.385	0.81 (0.26–2.53)	0.720
Age (year)		1.21 (1.08–1.35)	0.001	0.80 (0.73–0.88)	< 0.001
Female gender		0.16 (0.03–1.08)	0.060	1.42 (0.99–2.02)	0.055
Age * Sex (Girl)		1.21 (1.02–1.44)	0.031	–	–
Urban residence		1.75 (1.15–2.65)	0.009	0.94 (0.62–1.43)	0.786
Outdoor activity time (hour/day)		0.999 (0.997–1.001)	0.314	0.999 (0.996–1.001)	0.339
Time spent for watching TV and digital games (hour/day)		1.001 (0.999–1.003)	0.228	1.002 (1.000–1.004)	0.052

*CI* confidence intervals.

The logistic regression results for the association between three scale iris colors and refractive errors were not significant.

## Discussion

This study showed that while dark brown is the most common iris color in Iranian students, there is no evidence to support any association between the iris color and hyperopia.

However, myopia is more probable in students with amber than dark brown iris colors. Other studies on children^12^ and adolescents^27^ have reported a higher prevalence of myopia in darker irises. Studies on the association between refractive errors and iris color are few so in a review study conducted in 2014, no study investigated the association between iris color and myopia^28^.

This study also revealed that the axial length in different groups of iris color was not significantly different. However, studies that reported more myopia in a darker iris color also found that the axial length was larger in this iris color than in other colors^12^.

One hypothesis to explain the difference between this study and other studies is that the diagnosis of myopia may be based on non-cycloplegic refraction or that cycloplegia may be incomplete in children with darker iris color, leading to the overestimation of myopia in these children. Since melanin is a polyanion with a synthetic affinity for cationic substances (metals and amines), it combines with cycloplegic agents (muscarinic antagonists). Therefore, achieving complete cycloplegia in children with darker irises is more difficult than in children with lighter eye colors.^12^ Previous studies have also shown that the prevalence of myopia in non-cycloplegic conditions is higher than in cycloplegic conditions^29^.

On the other hand, the results of the present study are almost similar to the results of another study carried out in Iran, which indicated that people who had a lighter iris color (green/yellow) had higher odds of myopia than people with dark brown iris color^16^. In these two studies, in addition to the differences in how the iris color was classified, the age of the participants and the geographical area were also different. Finally, the difference in the prevalence of refractive errors and iris color in different studies can be a reason for the difference in the above-mentioned results.

It should be noted that genetics is considered the main factor in determining the color of the iris^30^. However, environmental factors also interact with iris color directly or indirectly, which can be due to filtering a particular color or wavelength^6,30^. It is hypothesized that iris color is associated with myopia by different filtering rates of light and its wavelengths^6,12^. The iris has two types of melanin called eumelanin and pheomelanin. Eumelanin forms the color spectrum of brown to black, and pheomelanin forms the color spectrum of yellow to red. Eumelanin absorbs light at longer wavelengths more than pheomelanin. Naturally, the more eumelanin the iris has, the more light and energy it absorbs than pheomelanin, and the higher the temperature of the iris and surrounding tissues will be^31,32^. Iris color can affect the development of myopia because the type of melanin, that determines iris color, causes different wavelengths to pass through the eye and ultimately affects the growth of eye parameters such as axial length and refractive power. In fact, the growth rate of the eye and subsequent refractive errors are influenced by the amount of light filtered by iris pigments and melanins^6,12^. Another study showed that children with more exposure to artificial light at night during infancy had more premature myopia^33^.

The present study shows that myopia was more probable in urban area. This finding is in line with other studies^33–35^ and can be attributed to more outdoor activity and less near work times in rural residents, although these factors were not associated with myopia and hyperopia in this report and another study^36^. Although different and inconclusive results have been reported regarding the relationship between outdoor activity and near work time with myopia^37–39^, recent cohort^40^ and randomized clinical trial^41^ studies confirmed these variables as influencing factors for the incidence and progression of myopia.

The large sample size, cycloplegic refraction, and careful examination with continuous monitoring were among the strengths of this study. However, it has a few limitations including the predominance of one iris color (dark brown) and the low prevalence of other colors (including amber color) in the studied population, not investigating the genetic status and polymorphisms associated with iris color, and subjective classification of iris colors. The prevalence of hyperopia and myopia in the study population was relatively low, and in some iris color groups, there were a small number of students with hyperopia or myopia, which should be considered as another limitation. It should also be noted that, no causal inference can be made from the results of this cross-sectional study, even though the color of the iris can be assumed to be unchanged, and to some extent, temporality has been established in this study. Finally, we did not measure the inter or intra-examiner agreement in classification of iris colors, which may be other limitation of current study.

## Conclusion

Our study suggests that iris color has no association with hyperopia, but amber color can be considered as a risk factor for myopia. Considering sparse data in some iris colors, further studies with larger sample sizes as well as longitudinal studies are necessary to evaluate this conclusion and the incidence of myopia in each of the iris colors.

## Data Availability

The data that support the findings of this study are available from the corresponding author, [MHE], upon reasonable request.
